# Multimodal Worlds, Multilingual Selves: Fictional Linguistic Landscapes in Transnational Education

**DOI:** 10.3390/bs16030450

**Published:** 2026-03-18

**Authors:** Osman Solmaz

**Affiliations:** Department of Foreign Language Education, Dicle University, Diyarbakır 21280, Türkiye; osolmaz@dicle.edu.tr

**Keywords:** transnational youth, fictional linguistic landscapes, multilingualism, fan practices, linguistic landscapes

## Abstract

Transnational youth frequently navigate multiple languages and continually negotiate not only affiliation, but also the legitimacy of the languages they use within changing linguistic hierarchies. However, their educational experiences are often framed through fragmented classroom practices, deficit-based assessments, and nationally bounded curricular frameworks. In this paper, I respond by theorizing Fictional Linguistic Landscapes (FLL) as a transdisciplinary pedagogical approach that utilizes fiction and participatory cultural practices to position language learning as a form of semiotic design, critical inquiry, and identity (re)work. Grounded in linguistic landscape studies, multiliteracies pedagogy, and fan-based meaning-making, FLL positions learners as world-builders and allows them to experiment with visibility, hierarchy, and language(s) in safe fictional environments. This study outlines the four-phase FLL in Second Language Teaching and Learning (L2TL) cycle and provides five pedagogical design spaces to address issues of raciolinguistic valuation, deficit institutional representations, affective harm, peer-level marginalization, and translocal or return migrant identity negotiation. Rather than viewing imagination as an outcome of teaching, FLLinL2TL structures it as a necessary process for learning, linking creative production to explicit linguistic objectives and reflective justification. I conclude by discussing implications for classroom practice, teacher education, and future research on the potential of the FLLinL2TL approach in transnational education research.

## 1. Introduction

Transnational youth now constitute a considerable presence in educational systems worldwide, moving daily among multiple languages, cultural affiliations, and geographic contexts. As a result of their mobility, they engage with multiple languages, cultures, and geographies on a daily basis. Rather than experiencing their linguistic and educational realities based upon national identities, international youth experience their realities through their movement, displacement, and their trans-local connections across borders. Although transnational youth have been framed through narratives of disadvantage and limited ability, research has shown that transnational youth are able to move freely across identities and social roles (Frausto-Hernandez, 2025). Similarly, research concerning transnational youth demonstrates that they negotiate their identities across disciplinary boundaries and at varying educational levels (Tharapos & O’Connell, 2022). However, pedagogical engagements with transnationality continue to be fragmented, deficit-oriented, and narrowly framed. In this article, I aim to address the disconnect described above in order to rethink pedagogical engagements with transnationality and focus on the semiotic and experiential realities of transnational youth.

Transnational youth continue to struggle with certain structural barriers throughout both their linguistic and educational experiences. Their multilingual repertoires are frequently restricted or devalued in educational settings in which particular languages, standard forms, and monolingual norms are privileged (Jiang, 2025; Miao & Yang, 2024). Language ideologies such as these are not neutral and are informed by racialized assumptions that position transnational youth as deficient and vulnerable rather than multilingual and resource-rich (Frausto-Hernandez, 2025; Zhang-Wu et al., 2024). These frames have implications for how learners develop their linguistic funds of knowledge across borders. In addition, the frames reinforce ideologies such as native-speakerism that view their repertoires as diminishing rather than enriching (Mora-Pablo et al., 2025). As a result, learners’ everyday linguistic practices are framed as problems rather than as linguistic resources for meaning-making (Herrera, 2023). Additionally, these dynamics operate affectively by creating shame regarding learners’ names, accents, and cultural histories, which requires learners to suppress their identities in pursuit of academic legitimacy (Jiang, 2025).

In many educational contexts, curriculum misalignment contributes to the marginalization of transnational learners because curricular frameworks often remain centered on national narratives and relatively static cultural representations that do not fully reflect the transnational realities many learners experience. Instructional resources may therefore overlook learners’ experiences of mobility, provide limited representations of hybrid identities, or fail to meaningfully engage with the diverse linguistic and cultural trajectories present in contemporary classrooms. At the same time, the extent of such misalignment varies across educational systems, particularly in regions where transnational mobility and multilingualism are already embedded within curricular traditions. For returning and transnational learners, this disconnect becomes visible when their linguistic trajectories fall outside the narrow definitions of academic language typically recognized in schooling (Tacelosky, 2018; Villegas-Torres & Mora-Pablo, 2018). Therefore, regardless of proficiency levels, learners may be positioned as linguistically incompetent, requiring them to be agents of their own identity negotiations, and resist raciolinguistic positioning in order to create legitimacy in academic contexts (Zhang-Wu et al., 2024). Although, it is also significant to note that schooling institutions rarely create ongoing pedagogical environments where learners are able to develop emergent identities, imagine alternative futures, and define belonging outside of pre-prescribed contexts. In such circumstances, when transnational backgrounds are unacknowledged, learners experience gaps in belonging on an everyday basis, as they attempt to navigate the fragmented identities they develop in educational systems that view their transnational knowledge as illegitimate (Frausto-Hernandez, 2025).

Despite the growing scholarship on language policy, identity, socialization, and power in transnational education, the role of fiction and imagination has received comparatively little conceptual attention. Existing approaches tend to emphasize adaptation to institutional systems, navigation of real-world constraints, and documentation of lived realities. While these perspectives have played an essential role in making inequities visible and documenting learners’ lived experiences, they tend to center the ongoing work of navigation and survival within already established borders. In doing so, they leave comparatively limited pedagogical space for approaches that invite learners to reconfigure those structures, rather than simply endure them (Frausto-Hernandez, 2025). Although prior research shows how transnational youth navigate linguistic and social constraints often through digital and translingual practices (Skerrett, 2020; Mora-Pablo et al., 2025; Zhang-Wu et al., 2024), it tends to focus on how learners adapt to existing conditions. As Kim (2018) notes, educational research has paid relatively little attention to pedagogical approaches that position learners as designers of social futures rather than simply as navigators of inherited structures. When imagination is acknowledged, it is often treated as secondary and valued as creative enrichment or extracurricular activity rather than as a core epistemological resource. Yet research on digital and transnational literacies indicates that learners already engage in imaginative, multimodal meaning-making beyond the classroom, mobilizing full semiotic repertoires to negotiate identity and belonging (Kim, 2018). Focusing only on real-world physical linguistic landscapes and authentic contexts risks overlooking the generative potential of fictional and speculative spaces, where learners can explore power, identity, and language without the immediate social constraints of real-world stratification (Gorter & Cenoz, 2023; Solmaz, 2025). The continued under-theorization of imagination within formal education thus limits how transnational education conceptualizes agency, creativity, and future-oriented learning.

Against this backdrop, I suggest that fiction, including literary, film, and other narrative worlds, can be viewed not as a secondary activity, but as a method for conceptualizing imagination as a core pedagogical resource. The indirect nature of fictional worlds creates conditions for learners to examine sensitive issues and power relations while exploring identity positions, functioning as protective semiotic spaces that facilitate agency and risk-taking (Solmaz, 2025). Because transnational literacies already operate across language, art, movement, and narrative, fiction becomes a particularly generative site for pedagogical legitimation (Skerrett, 2020). Narrative and multimodal storytelling have been shown to function as anti-racist pedagogies that help learners confront linguistic shame and resist internalized hierarchies (Jiang, 2025), while fictional storytelling also enables classrooms to operate as additional spaces for imagining belonging in home and host contexts. Research on fan-based and affinity spaces further demonstrates how fictional and participatory cultures support translocal identity formation beyond geographic constraints (Sya et al., 2022), highlighting fiction’s capacity to reframe whose voices, languages, and futures are recognized in education.

In this article, I introduce Fictional Linguistic Landscapes (FLL) as both a pedagogical framework and a conceptual lens that shifts attention from observing semiotic environments to authoring imagined ones. Building on linguistic landscape (LL) studies, multiliteracies pedagogy, and fan-based and speculative practices, FLL conceptualizes LLs as ideologically saturated spaces embedded in histories, power relations, and struggles over visibility rather than neutral representations of language use (Pennycook, 2017; Shohamy & Waksman, 2009). This shift redefines learners as designers, authors, and world-builders and treats language as a semiotic resource and ideological choice through which identities are shaped across real and imagined worlds. Through FLL, transnational youth can co-construct semiotic worlds that foreground multilingual repertoires and hybrid identities as sites of agency rather than remediation (Solmaz, 2025). This framing positions FLL as a pedagogical and epistemological shift that prioritizes design, critical inquiry, and sociolinguistic awareness in ways that connect fictional contexts to real-world applications (Solmaz, 2025).

The purpose of this article is to outline potential pedagogical design spaces for transnational youth using Fictional Linguistic Landscapes (FLL) and to illustrate how it may assist in addressing the persistent challenges of transnational education. I take a conceptual approach rather than an empirical one, and present directions for both practice and future research. The guiding question is: What new possibilities arise when transnational learners design worlds rather than simply navigate existing ones?

## 2. Conceptual Foundations of Fictional Linguistic Landscapes

### 2.1. Linguistic Landscapes as Ideological and Semiotic Spaces

Linguistic landscapes (LLs) have been described by increasing numbers of researchers as “meaning-making” environments as opposed to simply being a reflection of the languages used in the environment. From the “meaning-making” perspective, the semiotic resources located in public space affect social relationships, identities, and power dynamics as well as define how languages are viewed, evaluated, and legitimized (Przymus et al., 2025; Solmaz & Przymus, 2021). Accordingly, LLs are not merely a background against which we observe the world of language; they contribute to the creation of social reality through the normalization of specific hierarchical structures of language and specific forms of recognition (Przymus & Solmaz, 2025).

This conceptualization is further supported by scholars who view LL as multimodal, multisensory semiotic assemblages that extend beyond written language to include visual, spatial, auditory, and interactive resources (Shohamy, 2019). Within these types of assemblages, visibility becomes ideologically significant. LLs are therefore “far from neutral,” and index the results of negotiated relations of legitimacy, authority, and symbolic power (Pennycook, 2017). Prominence functions as an indicator of belonging and authorship; lack of prominence, and/or marginal placement, indicates exclusion and delegitimization (Gorter & Cenoz, 2023). Thus, LLs function as regulatory mechanisms that determine what is displayed in public space and can be viewed as surface expressions (i.e., “the tip of the iceberg”) of deeper historical, political, and ideological processes that develop and change language hierarchies over time (Shohamy & Waksman, 2009).

This regulation is especially visible in signage, which, through its ubiquitous display of commercial and institutional texts, plays a major role in organizing public space. The regular occurrence of such signage can contribute to the reproduction of national visions of identity, the standardization of linguistic ideology, and the promotion of monolingualism, although other linguistic landscapes may instead highlight bilingual or multilingual practices and challenge dominant language hierarchies. In doing so, these semiotic displays establish implicit regimes of value that assign social status to particular languages and their speakers (Shohamy, 2019).

What remains less explicitly addressed in this body of scholarship, however, is transnationality. Despite the fact that transnational aspects are often indicated when discussing hybridity, mobility and trans-local repertoires, they are often left out of the analysis, indicating the limits of national, bounded semiotic systems. I argue that these limits have particularly significant implications for transnational learners whose daily linguistic practices often extend beyond the semiotic boundaries established in public space. When semiotic space is viewed not only as a physical environment but also as an ideological arena where visibility, voice, and legitimacy are contested, the limits of what can occur within it become apparent. Therefore, by approaching LL as inherently ideological, identity-forming, and implicitly educational (Przymus & Solmaz, 2025; Shohamy, 2019), it may be possible to understand how the ideologies of the semiotic space impact the transnational learner’s experience of language learning.

### 2.2. Pedagogical Linguistic Landscapes

Pedagogy-oriented LLs have emerged as a strand of LL research that integrates everyday semiotic environments into language education, repositioning public space as a site of learning rather than a passive instructional backdrop (Gorter & Cenoz, 2023; Krompák et al., 2022; Solmaz & Przymus, 2021). It is commonly organized around a dual orientation: learning in the LL through direct engagement with public space, and learning through LL via pedagogical recontextualization of LL data. Together, these orientations bridge classroom instruction with lived linguistic ecologies and foreground language as socially situated and ideologically mediated rather than neutral or purely functional (see Melo-Pfeifer, 2023).

Empirical research shows that pedagogical LL can lead to greater sociolinguistic awareness and critical understanding of how people use language and create their own power relationships and language ideologies (Malinowski & Dubreil, 2019), and to increased engagement of learners and to critically observe semiotic elements in authentic environments (Gorter & Cenoz, 2023). In addition to increasing awareness and encouraging students to become active participants in analyzing and creating their own semiotic environments, it also provides students with opportunities to engage in public semiotic space in a way that will allow them to analyze, critique, and recreate these environments (Krompák et al., 2022; Melo-Pfeifer, 2023).

Although there are many LL-oriented pedagogical practices, most are still analytical, providing students with opportunities to focus on aspects of the semiotic order they inherit (e.g., noticing, documenting, interpreting), while offering students limited opportunities to actively reshape these environments (Przymus et al., 2025; Solmaz, 2025). This lack of agency may limit the transformative potential of pedagogy-oriented LL approaches (Przymus et al., 2025).

The Linguistic Landscapes in Second Language Teaching and Learning (LLinL2TL) model (see Solmaz, 2021) is an example of a pedagogical approach that integrates multiliteracies and multimodalities into a single model, and includes both reflection and transformed practice, which allows for multiple forms of semiotic diversity in both physical and digital spaces. The development of symbolic competence within this framework enables students to interpret the ideological meanings behind different semiotic messages and representations, and to reformulate these messages and representations themselves in the public sphere (Przymus & Solmaz, 2025). However, even though the LLinL2TL model has a focus on transformed practice, the focus on design implies creative transformation of semiotic elements, but does not imply sustained authorship, world making, or futurity (Solmaz, 2025). In particular, I argue that this limitation is especially relevant for transnational learners who already make meaning from a variety of languages, modalities, and environments every day. However, because they are positioned as primarily interpretive in the classroom, they may not have opportunities to explore design as an aspect of transformed practice.

### 2.3. Fan Practices, Fiction, and Imagination as Pedagogical Experiences

Fan practices offer a rather contrasting model, constituting participatory literacy ecologies in which multilingual and multimodal meaning-making is central rather than peripheral to literacy development (Solmaz, 2025). Practices such as fan fiction, fan art, cosplay, and fan translation blur the boundary between consumption and production, positioning participants as cultural authors engaged in collaborative redesign rather than as passive recipients of meaning (Black, 2009; Jenkins, 2006; Sauro, 2020). Literacy, in this context, is enacted through participation, negotiation, and authorship, thus creating an opportunity to question and challenge the distinction between formal and informal learning, and to think about the new ways literacies are conceptualized.

Beyond participation and authorship, fan and affinity spaces create sites for translocal belonging, which allow fans to negotiate their identities, languages and affiliations across geography, culture, and language boundaries (Jenkins, 2006; Stein & Busse, 2009). Although other fictional or creative writing practices may offer similar opportunities for identity exploration, fan spaces also foster transcultural and translingual practices, which can help us to understand how people learn to live in multiple cultures and languages, and who do not learn to live in one country, or in one language (Black, 2009). Therefore, in terms of how people learn to make meaning, fan spaces reflect the lived reality of many transnational youth, whose everyday lives already take place across borders, modes, and languages.

Within these translocal and participatory spaces, fiction occupies a central role in fan spaces because it allows fans to engage indirectly with sensitive topics by using the distance provided by fiction rather than the immediacy of self-disclosure (Black, 2009). This distance creates a space for taking risks and experimenting with different identity positions and social relationships, without the immediate real-world identity constraints. Examples of how fans use fiction to experiment with identity include alternate universes, gender-swapping, and race-bending which all demonstrate how fans use fictional reconfiguration as a semiotic resource to imagine and reimagine power, belonging, and subjectivity (Sauro, 2020).

While fans may be engaging in these experiments with identity, they are still developing complex literacy practices across genres, audiences, and media (Sauro, 2020), which challenges the traditional definition of what is meant by academic literacy. The imagination within these practices is closely connected to critical engagement with power and ideology, as they reinterpret canonical texts, challenge dominant meanings, and reconfigure cultural narratives in order to imagine a future that is full of possibilities beyond the constraints of formal institutions (Solmaz, 2025). In this sense, fan literacies implicitly critique formal schooling’s emphasis on correctness, standardization, and compliance over voice, authorship, and epistemological engagement (Sauro, 2020).

Research in pedagogy-oriented LL has started to feature imagination, redesign, and symbolic play, particularly through ‘transformed practices’. However, the research usually treats these elements as techniques or results of an educational process, rather than as structured learning environments. LL-based pedagogical activities that incorporate practices such as documenting, analyzing, and creatively redesigning public signage demonstrate imagination’s pedagogical potential but often treat it as a technique or outcome rather than as a sustained condition for learning (see Gorter & Cenoz, 2023). This gap between imagination as method and imagination as infrastructure points to the need for a reconceptualization in which fiction and fan practices are understood as structured learning itself, thereby pointing toward Fictional Linguistic Landscapes (FLL) as designed, speculative, and agency-oriented pedagogical spaces (Solmaz, 2025).

Overall, these findings highlight the limits of pedagogical approaches that privilege analysis over design in LL work, a concern raised in recent educational LL research (Przymus et al., 2025; Przymus & Solmaz, 2025). Fan practices and fictional meaning-making show how learners already engage in semiotic redesign, world-building, and future-oriented authorship beyond institutional contexts. In the following section, I introduce Fictional Linguistic Landscapes (FLL) as a framework that reconceptualizes imagination as pedagogical infrastructure rather than enrichment.

## 3. Fictional Linguistic Landscapes as a Pedagogical Model

### 3.1. Defining Fictional Linguistic Landscapes as a Model

I propose the concept of Fictional Linguistic Landscapes (FLL) as a pedagogical model that combines aspects of fan practices and linguistic landscape (LL) studies to develop literacies-oriented language learning experiences. The FLLinL2TL (Fictional Linguistic Landscapes in Second Language Teaching and Learning) model incorporates fan-based activities such as fan fiction, fan art, and world building with the pedagogical LL practices. Learners no longer only analyze semiotic environments but create fictional ones as well (Solmaz, 2025). This is also consistent with literature on fan practices that has moved from viewing fans as simply interpreting texts to viewing them as actively creating meaning and therefore as both interpreters and authors (Stein & Busse, 2009). Therefore, I argue that FLL shifts the way we view LL pedagogy, which is typically understood as pedagogical approaches in which students analyze and interpret public signs and semiotic resources, from the interpretation of pre-existing signs to the creation of semiotic systems to produce meaning, to shape visibility, and to distribute power (Solmaz, 2025).

In FLL, there is a transition from documenting to world building, which positions learners from being passively observing an established semiotic system to designing internally coherent semiotic environments (Solmaz, 2025). The principles of this transition can be found in fan practices that demonstrate how meaning-making moves from receiving a message to creating it and from analyzing a message to collaboratively expanding semiotic worlds (Jenkins, 2006; Stein & Busse, 2009). Therefore, I suggest that fictional spaces can serve as analytic sites that allow learners to experiment with semiotic choice, hierarchy, and representation in ways that are not constrained by existing socio-political orders. As such, through activities such as creating fictional signage, designing symbolic systems, and developing narrative worlds, language ideologies and power relationships become visible and subject to critique (Solmaz, 2025).

Pedagogically, I have structured the FLL model around four interconnected phases (i.e., Situated Practice, Guided Exploration, Creation and Transformed Practice), which provide a structure to integrate fan practices and analysis of semiotic landscapes into language instruction (Solmaz, 2025). Learners progress from engaging with familiar cultural texts toward critically and creatively constructing semiotic landscapes as they reflect upon how language, symbols and modes operate in fictional and real-world contexts. This progression mirrors research on fan practices indicating that learning emerges through participation, collaboration and shared semiotic problem solving (i.e., collectively making decisions about meaning, language use, symbols, and representation across different modes), and not through a linear progression of developing skills (Sauro, 2020). While pedagogical LL approaches that previously legitimized semiotic production primarily at the level of real-world signs, I contend that FLL expands the range of learner authorship and agency to the level of world building.

A central feature of the FLL model is its principle of maintaining a balance between linguistic practice and creative expression. While imagination is fundamental to generating interest in the activity, I argue that FLL requires creativity be grounded in clearly defined linguistic objectives so that the process of meaning-making contributes directly to language development rather than providing supplemental enrichment (Solmaz, 2025). The practical implementation of this balance is accomplished through structured creativity, which links fan-based activities such as designing signage, writing captions or producing fictional social media texts to specific language skills and communicative functions, thereby requiring sustained focus on form, meaning and use. Based on social semiotic theories that conceptualize sign-making as selecting and combining existing resources, FLL uses scaffolding techniques including examples, gradual increases in semiotic complexity, peer interaction and iterative feedback (Solmaz, 2025).

Together, these elements define FLL as a multimodal and interdisciplinary model that places L2 learning within participatory culture (Jenkins, 2006) and semiotic design. By defining learners as world builders who have the capacity to construct their own linguistic and cultural realities, FLL extends LL pedagogy to include authorship and micro policy-making within designed worlds, where multilingualism is seen as dynamic, symbolic and translocal rather than territorial (Solmaz, 2025). Therefore, I argue that FLL serves as a pedagogical framework, a conceptual lens and an epistemological intervention to redefine how language, power and identity are engaged in educational settings.

### 3.2. The Four Phases in Practice

**Figure 1:**
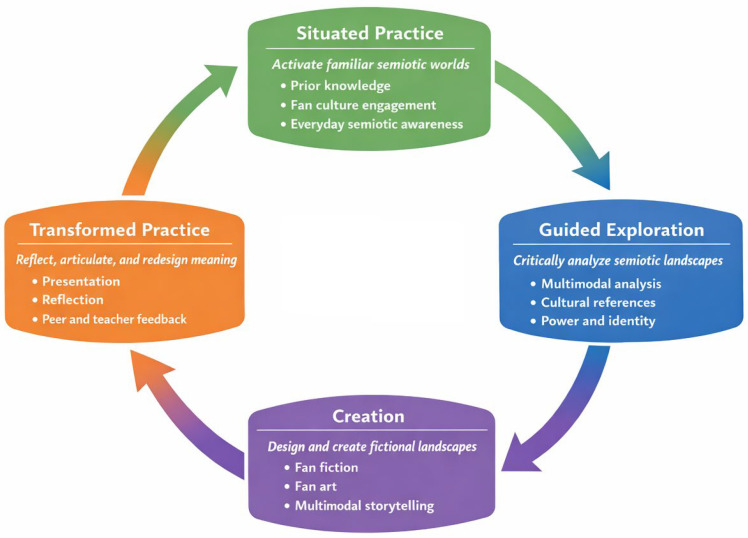
The Fictional Linguistic Landscapes in L2 Teaching and Learning Pedagogical Cycle.

I have grounded the four-phase structure of the FLLinL2TL model in literacies-oriented pedagogies that conceptualize learning as situated, socially mediated, and design-driven (see Figure 1). Rather than functioning as a linear sequence of activities, I propose that the four phases of Situated Practice, Guided Exploration, Creation, and Transformed Practice function as an integrated pedagogical cycle that supports learners’ movement from engagement with familiar semiotic resources toward critical and creative meaning-making across fictional and real-world contexts (see Solmaz, 2025 for a comprehensive explanation).

In the ‘Situated Practice’ phase, instruction begins by connecting new content to learners’ existing knowledge, emotional investment, and experiential familiarity with fan culture and semiotic landscapes. Using recognizable media texts and everyday semiotic environments, this phase enables learners to recognize themselves as knowledgeable participants whose prior engagements with language and symbolism are viewed as pedagogical resources, and not as a distraction. By linking abstract linguistic concepts to the lived and imagined experiences of learners, educators can establish the conditions for learners to begin reflecting on how signs, symbols and modes of communication convey meaning in context.

Building upon this situated engagement, the ‘Guided Exploration’ phase transitions learners from participation to analysis. During this phase, learners compare fan-generated artifacts with source texts, and examine how meaning is created across semiotic landscapes in both fictional and real-world contexts. Educators utilize a variety of multimodal resources (e.g., images, videos, posters, and digital platforms) to make the semiotic processes involved in creating meaning. As a result, learners have the opportunity to critically examine how language, visual and spatial arrangement create references to cultural values, identities and power relationships. While linguistic development is still a primary objective of this phase, I emphasize that it also focuses on developing critical literacies by providing learners with opportunities to analyze the ideological and symbolic dimensions incorporated into both fan-produced artifacts and other LL materials.

The next phase, ‘Creation’, represents the most significant design-oriented aspect of the model. In this phase, learners actively participate in fan practices such as fan fiction, fan art, and multimodal digital creation to develop their own fictional semiotic landscapes. Utilizing Jenkins’ (2006) concept of participatory culture, learners collaborate to create worlds, characters, and narratives using a wide range of linguistic and semiotic resources, both across modes and through various platforms. This creative activity allows learners to be seen as authors of meaning, and not simply as consumers of it, and the collaborative nature of this activity along with educator support and peer feedback ensures that the creative output is linguistically and pedagogically meaningful. I conceptualize this as an example of semiotic repurposing, where existing texts and symbols are recontextualized to create new meanings and functions.

The ‘Transformed Practice’ phase provides a reflective component to the cycle. In this phase, learners formally present and justify their creative outputs, and explicitly articulate the semiotic choices, linguistic strategies and design decisions they made when creating those outputs. Through the use of peer and teacher feedback, learners can further understand how meaning is created across fictional and real world semiotic landscapes, and through reflective activities, learners are invited to explore how their identity, cultural affiliation and experience(s) influenced their interpretations and creations. Furthermore, I advocate for flexible forms of assessment that consider both the process and product of the learners’ creative output to enable the development of linguistic competence in addition to critical thinking, creativity and collaboration, while also emphasizing the model’s focus on learning as transformation and not reproduction.

Finally, I find it important to note that these phases can be recursive and adaptable, and thus, educators can emphasize specific aspects based on instructional objectives, student characteristics and institutional contexts.

### 3.3. Pedagogical Affordances and Potential Challenges

I argue that Fictional Linguistic Landscapes (FLL) offer a rich environment for creating opportunities for multimodal and literacies-centered language learning, but there are certain instructional and contextual challenges associated with implementing it that require critical consideration. I propose that the model provides an overarching pedagogical framework to facilitate language development through multimodal and experiential learning processes and create spaces for learner collaboration and creative expression. It enables learners to engage critically with issues related to power, ideology and sociocultural aspects of language use as well as how language is used to construct and contest meaning in both fictional and real world contexts. Furthermore, FLL-based practices can contribute to multiliteracies through the integration of digital, cultural, and intertextual resources, thereby connecting the learning of students within the classroom to their out-of-school participatory meaning-making activities. In addition, I contend that the model provides teachers with a structured but flexible base for developing culturally responsive teaching practices.

However, although the model creates a dynamic process for language development, I recognize that educators need to balance between the creative elements and the explicit linguistic developmental goals of this approach. Educators should also be sensitive to the cultural backgrounds of all participants and deal with the possible reluctance of educators to engage with fan culture and/or the lack of knowledge about fan culture on the part of some teachers. Finally, educators need to manage the limitations of time and resources when attempting to implement it.

## 4. FLL-Oriented Pedagogical Design Spaces for Transnational Youth

In this section, I present pedagogical implications of Fictional Linguistic Landscapes (FLL) for transnational youth in this section. In particular, I see FLL as a design-based pedagogical framework to respond to the ways in which issues of legitimacy, belonging, and voice are contested in the context of educational spaces characterized by inequality, exclusion, and misrecognition. In contrast to decontextualized instructional approaches, FLL reorients pedagogy toward authorship, imagination, and semiotic agency by inviting learners to construct fictional multimodal worlds through which language, identity, and power can be critically explored. Highlighting imagination and semiotic agency, FLL enables learners to create fictional, multimodal worlds that serve as platforms for critical examination of language, identity, and power.

The pedagogical examples I present below represent only a sample. My goal is to demonstrate how FLL-oriented practices can be implemented across the four phases of the model in flexible and context-sensitive ways. The level of abstraction, linguistic complexity, and modes of representation can be adapted across age groups, proficiency levels, curricular demands, and institutional constraints. For instance, students at a lower level of proficiency may have opportunities to explore FLL through multimodal experiences (e.g., visual storytelling, character design, short narratives) that are supported by a teacher/guide. On the other hand, students at an upper level of proficiency may complete extended narrative writing, critical semiotic analyses of multimodal texts, or complex multimodal production. In this way, the framework is not limited to advanced language learners but can be scaled to different proficiency levels by adjusting the linguistic complexity and degree of analytical depth expected in each phase. What remains central across these design spaces is a shared pedagogical shift. Instead of evaluating learners’ language against established norms, the focus moves towards engaging them as designers of semiotic systems in which language, power, identity, and belonging are made visible. Rather than seeking to resolve raciolinguistic, institutional, or affective inequities directly, FLL provides structured opportunities for learners to examine how such conditions are produced and to reimagine the social, institutional, and symbolic structures that define linguistic legitimacy.

The phases of the FLL model are designed for use in classrooms as an adaptable and flexible cycle of teaching. They should not be viewed as a sequential process with a specific number of classes or time devoted to each phase. The amount of time devoted to each phase may vary based upon the teacher’s choice of instructional strategies and methods, the amount of time available for instruction, and the needs of the students. For instance, some projects/units may involve only one or two classes for *Situated Practice* and/or *Guided Exploration*, followed by more extensive opportunities for students to engage in *Creation* and later in *Transformed Practice*. The flexibility of the model provides teachers the opportunity to adjust the length of time devoted to each phase of the model to meet their own instructional needs and to fit the curriculum of their school, district, or region. Additionally, it allows teachers to provide a continuous, progressive sequence of learning experiences to their students; however, this does not have to be an experience that is always completed in a linear manner. Students may be able to work independently and simultaneously complete tasks at different levels of the model. Finally, assessment within FLL-oriented activities may combine conventional language-learning criteria (e.g., clarity of expression, vocabulary use, and communicative effectiveness) with broader dimensions of semiotic design, such as how learners represent identity, voice, and social positioning through linguistic and multimodal choices.

### 4.1. Responding to Raciolinguistic and Political–Economic Regimes of Linguistic Value

One of the common challenges reported in the literature is that raciolinguistic ideologies and political–economic regimes of linguistic value shape how transnational youth are positioned within educational spaces by regulating whose language is recognized, legitimized, and rewarded. Learners’ linguistic practices are frequently evaluated through the ‘white gaze’ and ‘native-speakerist’ norms, where value is assigned according to closeness to standardized English and, in some cases, its perceived market usefulness (Jiang, 2025; Smith, 2019; Zhang-Wu et al., 2024). Within such regimes, transnational learners’ repertoires are viewed as deficient regardless of communicative effectiveness, compelling students to strategically conform to dominant norms while managing ongoing experiences of exclusion and misrecognition (Herrera, 2023; Miao & Yang, 2024; Tharapos & O’Connell, 2022).

Rather than treating linguistic value as something learners must adapt to, I approach this challenge by inviting students to design semiotic worlds in which value itself is redefined. One example of implementing FLL-oriented pedagogy includes students working together to create a fictional city, community, or institution where multiple languages and varieties are considered as essential resources, as opposed to hierarchical exceptions. During the *Situated Practice* phase, educators can draw on familiar fictional worlds, for example, the fictional world of *Star Trek* and its ‘Universal Translator’ as an ideological artifact that shapes notions of intelligibility and power. Educators can also reference worlds such as ‘Wakanda’ from *Black Panther*, which is a world where multilingualism, symbolism and advanced technology coexist, and *Zootopia*, which represents institutional authority and access as being mediated through species-based differences that represent linguistic and social classifications. These shared semantic spaces are a practical starting point for thinking about how to produce visibility, audibility, and legitimacy in the public space.

Building on this engagement, the *Guided Exploration* phase can provide students with opportunities to think critically about how linguistic authority is distributed across signs, roles, and areas (real and fictional) and in ways that allow them to examine and compare different approaches to creating authority. Learners can also examine who is represented on public signage, what languages are associated with governance, mobility, or prestige, and how the inclusion/exclusion of particular groups is accomplished through translation, silence, or visual representation. Questions that can be explored during this stage could include: Who is assumed to need no translation; whose language needs to be mediated; which voices remain invisible even though they exist?

During the *Creation* phase, learners have the opportunity to design their own fictional semiotic artifacts (e.g., transportation systems, public notices, institutional policies or community guidelines) for their imagined city or institution. During this time, they are encouraged to experiment with alternative language hierarchies and assign value to language use based on criteria including narrative, relational, or cultural criteria as opposed to standardization or closeness to dominant norms. Examples of student designs could include designing multilingual transit signage that places emphasis on local knowledge, designing institutional policies that rotate linguistic authority among various groups, or developing symbolic representations that communicate meaning beyond written language.

Finally, during the *Transformed Practice* phase, learners reflect on the design choices they make in their fictional worlds and articulate how their worlds challenge, reproduce or complicate the dominant language ideologies. Their reflection may focus on questions such as: What is considered prestigious in this world? Who is audible by default? Who needs to be translated and why? How does the distribution of linguistic value in the fictional arrangement(s) mirror or disrupt the distribution of linguistic value in the real world educational and social environments? Through this process, FLL positions learners as not just analysts of linguistic inequality, but as designers capable of imagining alternative distributions of linguistic value.

### 4.2. Responding to Linguicism and Deficit-Based Institutional Framings

Another area of challenge is the continued impact of deficit-based representation of institutions that continue to influence transnational youth’s education experience by positioning their linguistic and cultural background as an obstacle to their ability to gain academic knowledge (Frausto-Hernandez, 2025; Mora-Pablo et al., 2025). Monolithic schooling models presuppose that students have standard language proficiency and linear, nation-bound trajectories of learning and therefore make the transnational students’ funds of knowledge invisible in curriculum, assessment practices, and classroom discourse (Tacelosky, 2018; Villegas-Torres & Mora-Pablo, 2018). Forms of linguicism operate in schools through both explicit language policies and everyday institutional routine that silence students’ multilingual repertoires and limit the conditions under which students can be recognized as competent participants (Herrera, 2023).

To conceptualize deficit-based institutional frames and make them visible and contestable, I use FLL as a theoretical space for redesigning educational institutions themselves. The pedagogical practice that exemplifies this concept has students design a fictional school and create its associated linguistic policies. This shift in focus enables an emphasis on institutional language ideologies as opposed to individual performances. In the *Situated Practice*, instruction utilizes students’ knowledge of well-known fictional schools. For example, *Hogwarts School of Witchcraft and Wizardry* (in the Harry Potter universe) can serve as an illustration of an institution with very specific and detailed institutional rules, and a high degree of semiotic density. Similarly, the *X-Men* series also serve as an example of schooling as a site of normalization, inclusion, and exclusion. Learners can analyze how institutional rules, hierarchies, and institutional texts create conditions for particular ways of speaking, participating, and being included/excluded.

The *Guided Exploration* phase supports student analysis of how institutional language functions across all types of policies, signage, curricular documents and assessment practices in both fictional and real world contexts. Learners may analyze how school rules, syllabi, disciplinary notices, etc., establish implicit standards regarding what constitutes legitimate language use, academic behavior, and acceptable identity. Attention can be paid to those moments in time when there are significant amounts of institutional control and reform, such as when Dolores Umbridge (the headmistress of Hogwarts School in *Harry Potter and the Order of the Phoenix*) imposes rigid rules and surveillance, to promote dialogue about how authority is established through language and how institutional texts can serve as instruments of exclusion.

The *Creation* phase provides opportunities for students to collaboratively work on fictional institutional artifacts (e.g., language policies, classroom guidelines, assessment rubrics, public documents) for their imagined schools. Through this process, students are encouraged to view linguistic diversity as a resource for participation and belonging, and not as something that needs to be managed. By engaging in design work, students are invited to question the foundational issues of institutional legitimacy: What constitutes “academic” language in this institution? Who establishes the norms of language usage in this school? How do multilingual and transnational practices get recognized, regulated, or erased in this setting?

Lastly, during the *Transformed Practice* phase, students articulate and justify their design decisions by reflecting on how their fictional institutions feature visibility, authority, and access in relation to the current educational systems in place. The focus of this reflection may include: alternative policies that challenge narratives of deficit; how institutional language can either limit or enhance participation; and what it means to imagine schooling beyond monolingual and nation-specific assumptions. Through this process, FLL positions students as not simply subjects of institutional norms, but as designers who can re-imagine how educational institutions define legitimacy, inclusion, and voice.

### 4.3. Responding to Affective Harm and Survival-Oriented Identity Negotiation

Beyond structural and institutional constraints, transnational youth experience emotional forms of harm that shape how they participate in language learning and negotiate identity in educational spaces. As documented in the literature, raciolinguistic ideologies and deficit narratives produce linguistic shame, accent phobia, and psychological vulnerability, compelling learners to hide aspects of their linguistic repertoires, adopt alternative identities, or strategically pass in order to avoid negative profiling (Jiang, 2025; Tacelosky, 2018; Zhang-Wu et al., 2024). These affective pressures result in survival-oriented identity negotiation, in which students balance the desire for academic safety with the need to affirm identity, often at significant emotional cost (Frausto-Hernandez, 2025; Smith, 2019).

When addressing affective harm and survival-oriented identity negotiation, I use FLL to highlight fiction’s capacity to create emotional distance without disengagement. One pedagogical practice can involve learners designing fictional characters or narrative worlds in which language choices, accents, and identities have social meaning. During the *Situated Practice* phase, instruction can draw on narrative worlds such as *Encanto*, where silence, voice, and legitimacy are negotiated within family and community relations in a fictional town in Colombia, and *Wednesday*, which centers marginal identities navigating institutional norms within a gothic setting in Vermont, USA. These stories can serve safe ways to explore how people use language, gestures, and multimodal design to express feelings of belonging, authority, and exclusion. In addition, students can analyze how fictional characters and semiotic environments use linguistic and paralinguistic choices to convey social status.

The *Guided Exploration* stage allows learners to reflect on how fictional characters and their semiotic worlds construct and represent social positioning(s) through their use of linguistic and paralinguistic features. As they do so, learners have the opportunity to consider such things as; who has permission to speak, when a character’s voice is silenced or misrepresented, how an accent or communication style is viewed as acceptable or unacceptable and how institutional/interpersonal expectations shape a character’s capacity to express their identity. Additionally, students can focus attention on how power functions at a micro level through tone, silences and gaze and how the emotional aspects of language marginalization are made visible.

In the *Creation* phase, learners create their own fictional characters, alternate egos, or worlds. Through the process of re-authoring a character’s voice or accent, altering a character’s background, or developing entirely new characters, learners explore identity positions that could be too difficult, or risky, to develop directly in the context of classroom participation. In this way, fiction functions as a protective semiotic barrier allowing learners to work with sensitive issues related to language, difference, and belonging. This phase focuses on the central question: What can this character say that I cannot? It is essentially a question that promotes the possibilities of expression without requiring learner exposure.

Finally, during the *Transformed Practice* phase, learners reflect on their creations, and articulate the symbolic, linguistic, and design decisions they made, while focusing on the process of meaning-making. Such reflection may include how characters gain or lose legitimacy, how voice is either limited or expanded, and how the fictional distance provides forms of expression that are often difficult to access within the boundaries of real-world classroom experiences. In this way, FLL positions fiction as an infrastructure for teaching, rather than as a means of escaping linguistic realities; and thereby provides learners with the opportunities to negotiate affect, identity, and agency with greater safety and intentionality.

### 4.4. Responding to Peer-Level Exclusion and Classroom Marginalization

Peer-level exclusion and classroom marginalization are critical dimensions of the educational experiences of transnational youth, as linguistic profiling and accent-based judgments are often expressed through everyday interactions rather than formal policies alone. As the literature indicates, learners may experience social isolation, bullying, or subtle forms of exclusion from peers who share their racial or cultural backgrounds but maintain dominant language norms (Mora-Pablo et al., 2025; Smith, 2019). These interactional dynamics reinforce raciolinguistic hierarchies in the classroom and place additional cognitive and emotional demands on students, as they need to monitor and regulate how they speak, participate and position themselves in relation to other students (Herrera, 2023; Tharapos & O’Connell, 2022).

In response to both peer-based exclusion and classroom-based marginalization, I envision FLL as a design space for restructuring participation through shared authorship. An example of an implementation would include small groups collaborating to design a fictional world, community, or narrative space in which the distribution of meaning is among all group members. Instruction in the *Situated Practice* phase may draw upon learners’ existing familiarity with collaborative storytelling cultures such as *Doctor Who* and its expansive and evolving canon based on contributions from many writers and fan communities, or *Dungeons & Dragons*, which situates collaborative world-building as the central aspect of storytelling processes. Both of these examples emphasize collaboration not as a supplemental skill but as a regular condition of meaning-making.

The *Guided Exploration* phase invites learners to examine how power, inclusion, and exclusion are produced within both fictional and real semiotic landscapes through collaborative processes. Learners may analyze how visibility and voice are distributed within shared narratives, whose contributions shape the evolving world, and how disagreement or difference is negotiated. Attention can be directed to how authority emerges through interaction rather than assignment, making visible the social processes through which meaning is co-constructed and legitimized. I use this phase to help learners recognize that exclusion is often not only the result of individual bias, but also of group dynamics and unexamined norms governing participation.

The *Creation* phase focuses on creating semiotic artifacts by groups of students in a collaborative setting; this can include developing narratives, public texts, information about the institution, and multimodal environments that require continued negotiation of both the linguistic and semiotic choices of all group members. As needed, groups can create assigned roles (e.g., narrator, language planner, visual designer, archivist) so that each member has structured support for their role in contributing to the project and not have to rely solely on informal group dynamics. Through this process, students can see how collaborative writing may produce different voices, challenge common participation patterns, and produce spaces where many of the same linguistic and semiotic repertoires can be used within the designed world.

The last phase, *Transformed Practice*, emphasizes that the focus should be on the process of how decisions were made, how differences were resolved, and how collaboration affected the final semiotic landscape. I suggest several questions, which may be used for reflection: How did collaboration redistribute voices? Who had their ideas visible, and why? What conditions allowed or limited the opportunity for students to participate? By making the collaborative writing process clear, FLL views collaborative writing as an approach to peer-level exclusion and classroom marginalization, and collaborative writing as a critical semiotic practice that produces a sense of belonging and legitimacy for its participants.

### 4.5. Responding to Translocal and Return Migrant Identity Negotiation

Translocal and return migrant learners often occupy identity positions that are difficult to accommodate within educational systems organized around monocultural, nation-bound assumptions. As documented in the literature, return migrants experience misrecognition and discrimination when their prior transnational experiences, linguistic repertoires, and backgrounds do not align with institutional expectations of linear language development or settled national belonging (Villegas-Torres & Mora-Pablo, 2018). These learners must navigate tensions between maintaining ethnic solidarity and meeting pressures to display standardized language proficiency in order to adjust themselves socially and academically (Miao & Yang, 2024).

I turn to FLL as a pedagogical site for translocal and return migrants’ identity negotiation. One activity may ask students to create fictional worlds (e.g., communities, institutions) based on mobility, liminality, and non-territorial affiliation. During the *Situated Practice* phase, instruction can utilize fictional worlds that represent survival spaces for multilingual displaced persons/refugees (i.e., *Children of Men*), identity shifts due to renaming/movement in/through liminal worlds (i.e., *Spirited Away*), and language as a transformative force for cognition/time/belonging beyond geographic boundaries (i.e., *Arrival*). Together, these alternatives provide imaginative ways to engage translocal experiences, without relying on nation-based frameworks.

Utilizing this as a foundation for the *Guided Exploration*, students can engage in examining the ways in which belonging, legitimacy, and voice are represented in mobile semiotic landscapes. Students may analyze how multilingual communication functions within uncertain contexts, how naming/re-naming operate as mechanisms for inclusion/erasure, and how language provides access to future possibilities/memory. This phase emphasizes that, although belonging can be established through spatial, symbolic and temporal relationships, this is not necessarily tied to the location itself.

During the *Creation* phase, students co-design fictional mobile worlds (i.e., borderless cities, nomadic institutions, borderland communities), in which language practices are influenced by circulation (of people). Students may map identities along routes or design institutions that do not have a fixed linguistic center. Through the creation of these fictional worlds, students can develop an understanding of how affiliation, legitimacy, and participation can be envisioned outside of stable national/institutional frameworks, while still focusing on linguistic form, meaning, and use.

The final phase, *Transformed Practice*, focuses on the reflective component of mobility as a semiotic condition. Students express how their fictional designs imagine belonging without borders, how language facilitates continuity across displacement, and how identity is (re)worked through movement. They may also reflect upon the following questions: What does belonging look like when a place is not permanent? How does language carry identity across worlds? By positioning mobility as a central component of design, FLL offers a pedagogical response to transnational and return migrant identity negotiation that goes beyond adapting to existing systems.

## 5. Conclusions and Implications

In this article, I conceptualize Fictional Linguistic Landscapes (FLL) as a design-based pedagogical framework that enables transnational youth to engage in world-building practices to reconsider participation, linguistic value, and institutional authority. Across the design spaces described above, fiction functions as a pedagogical foundation, making language ideologies visible, redefining ownership, and creating safe spaces for discussing issues of mobility, belonging, and legitimacy (Skerrett, 2020; Solmaz, 2025). By shifting critique and identity negotiation into imagined semiotic worlds, FLL allows learners to experiment with different forms of language and power that would be difficult or risky to address directly in real educational settings, especially for transnational youth positioned through deficit narratives (Frausto-Hernandez, 2025; Zhang-Wu et al., 2024).

Conceptually, the article bridges three bodies of scholarship that are generally covered separately: LL research, which emphasizes the ideological organization of semiotic space (Pennycook, 2017; Shohamy & Waksman, 2009); pedagogical LL work, where the recent focus on design still tends to center on the interpretation of existing signs (Przymus et al., 2025); and fan practices, where collaborative fiction already functions as a literacy base for multimodal authorship and translocal identity work (Jenkins, 2006). Synthesizing these strands, the article advances three interrelated conceptual outcomes: fiction for preserving semiotic distance, design for small-scale policy making, and world-building as identity (re)work.

Fictional Linguistic Landscapes carry distinct implications for pedagogy, teacher education, and research in transnational contexts. At the classroom level, FLL can have an impact on language teaching and pedagogy by having transnational students and teachers view legitimacy, inclusion, and multilingual development as a matter of design as opposed to being strictly bound by policy or compliance. With FLL, students are able to give authority and prestige to specific languages in fictional worlds, giving them the ability to develop their own critical language awareness through redesigning value systems rather than solely analyzing them. Fictional worlds offer safe environments where students can explore issues like accent, exclusion, and hierarchy without risking personal harm or vulnerability; while structured collaboration can create a sense of equity in participation.

Therefore, the nature of pedagogy changes from grading student work based on adherence to pre-defined standards, to encouraging students to engage in the process of designing their own semiotic systems in which language, identity, and power become visible and negotiated. I provide Appendix A (see below), which is a compiled list of optional design prompts, tied to the five FLL-oriented design spaces, which aims to provide adaptable ways for educators to implement FLL in their classrooms.

For teacher education, FLL supports a transition from instructional approaches that focus on accuracy, to those that are centered around semiotic noticing and design guidance, placing educators in the role of facilitating meaning-making as opposed to determining what is correct or incorrect. This, in return, requires educators to develop skills in recognizing and interpreting semiotic ideologies, to scaffold students’ creation of multimodal compositions while maintaining linguistic objectives, and to assess student learning through collaborative work, design rationale, and linguistic development. At the program level, the model provides educators with a modular approach to preparing students for multiliteracies, culturally sustaining pedagogies, and translanguaging-oriented models.

I recommend a research agenda that views FLL as a type of pedagogical model for researching language learning as a form of design, imagination, and social action. Future research may investigate interactional processes as transnational students build fictional worlds, particularly, how they interactively establish semiotic authority within the institutions they design. Discourse-analytic and ethnographic methods could be used in these studies to observe the emergence of authority through power, role distribution, and multimodal design decisions during world-building processes. In addition, longitudinal studies could examine how transnational students’ identity trajectories concerning voice, accent, and belonging are shaped over time by consistent engagement with these design practices. Attention to issues of equity and participation may further clarify the extent to which designs reconfigure power relations among multilingual students in the classroom.

While I do not present FLL as a prescriptive method, I propose a set of transferable implementation principles to support educators in adapting the framework in their own classrooms. I suggest that educators begin by selecting commonly shared fictional references, such as popular media worlds or narrative themes. This will help create a shared sense of “what we are working with,” and decrease potential access gaps created by differential levels of cultural capital. I suggest introducing the semiotic metalanguage (e.g., visibility, hierarchy, audience) early to enable students to engage reflectively and intentionally when creating their fictional landscapes. As such, I emphasize structured creativity, viewing genre, purpose, and audience constraints as supports for language learning. Educators can plan for equity in participation by providing students with clearly defined semiotic roles (e.g., policy planner, designer) so that students can share ownership and authority rather than assume it. Their processes can be assessed in conjunction with their products, focusing on the reasoning behind their decisions (e.g., justification), in addition to other factors (e.g., linguistic accuracy). Finally, ethical considerations must be taken into account, as well as protecting students’ well-being by maintaining the distinction between the fictional and non-fictional nature of the content they produce.

As a conceptual contribution, this study has several limitations that should be acknowledged. First, I propose a pedagogical framework and a number of design spaces, but I do not provide empirical data concerning whether or not the framework produces desired learning outcomes or improves instruction. Therefore, future research should investigate how the FLL approach functions in real-world classrooms across various educational contexts. A second limitation is that fictional references (especially those to popular franchise-based media) may replicate dominant, canonical, or exclusionary cultural narratives if educators who adopt the FLL approach do not diversify the range of fictional references used in the classroom. Therefore, educators who choose to implement the FLL approach need to pay attention to both representation and access issues. A final limitation is that there may be differences in students’ familiarity with fan cultures and fictional worlds. Unless educators take steps to scaffold students’ understanding of different genres and franchises, students and teachers unfamiliar with a particular type of franchise may experience barriers to participation in the activities of the course.

This article began by addressing persistent challenges in transnational education, including deficit-based representations of multilingual learners, disconnects between curricula and learners’ lived experiences, and emotional impacts associated with being linguistically marginalized. In response, I asked a guiding question: *What new possibilities arise when transnational learners design worlds rather than simply navigate existing ones?* I have argued that FLL offers one response by repositioning students as creators of semiotic systems rather than subjects of pre-existing linguistic structures. As students engage in fictional world-building, they can rehearse different ways of distributing voice, legitimacy and belonging while critically thinking about language, power, and identity. It is important to note that while these redesigns are imaginatively created, they also serve as the basis for the reflection upon, contestation of, and re-creation of actual educational spaces.

## Data Availability

The original contributions presented in this study are included in the article. Further inquiries can be directed to the corresponding author.

## Appendix A

These design tasks are intended to be adaptable resources rather than definitive activities. Depending on pedagogical goals, learner profiles, and institutional contexts, they may be selectively integrated.

**Table A1:** Selected Optional Design Tasks for Fictional Linguistic Landscapes.

Design Logic	Design Task	Pedagogical Focus	What the Task Makes Visible	Connection to FLL
Reconfiguring Linguistic Value	Designing zones of partial intelligibility in a fictional city (*not all messages accessible to all*)	Linguistic value, access, and power	How expectations of mutual intelligibility are unevenly distributed; who is expected to accommodate whom	Ideological critique, visibility/audibility in semiotic space
Institutional Legitimacy & Deficit Representations	Redesigning assessment criteria or academic rubrics for a fictional institution	Academic legitimacy	How “academic language” and competence are socially constructed and selectively rewarded	Authorship, transformed practice, redistribution of authority
Affective Protection & Voice	Designing alter-ego or parallel characters to explore voice, accent, or silence	Identity negotiation without self-disclosure	How fiction enables expression of risky positions safely	Protective semiotic space, imaginative agency
Collaboration & Distributed Authorship	Creating shared public texts (maps, notices) requiring negotiation	Collective meaning-making	How linguistic and multimodal choices emerge through dialog and compromise	Multimodality; socially mediated learning
Mobility & Translocal Belonging	Designing mobile or borderless institutions (schools, councils, communities)	Translocal affiliation	How legitimacy and participation can be imagined without fixed territorial centers	World-building; transformed practice
